# Focusing on legal cases: Automatic classification of legal documents with sentence embeddings and deep learning models

**DOI:** 10.1371/journal.pone.0350673

**Published:** 2026-06-05

**Authors:** Fawaz Khaled Alarfaj

**Affiliations:** Department of Management Information Systems, School of Business, King Faisal University, Al Ahsa, Saudi Arabia; Shifa Tameer-e-Millat University, PAKISTAN

## Abstract

The justice system is indispensable to any society as it enforces the rule of law, safeguards fundamental rights, and ensures the equitable resolution of disputes through structured legal frameworks. Artificial Intelligence (AI) has significantly advanced the legal and justice system by automating time-intensive tasks such as document review and contract analysis, thereby enhancing efficiency and reducing human error. Additionally, AI-powered predictive analytics and decision support systems have improved access to justice by providing data-driven insights, enabling faster case resolution, and ensuring more consistent application of the law. Legal document classification using AI techniques is imperative as it enables efficient organization, retrieval, and analysis of vast volumes of legal texts, enhancing accuracy, reducing manual effort, and facilitating faster decision-making in legal processes. In this research study, the main aim is to classify legal text documents using Machine Learning (ML) and state-of-the-art Deep Learning (DL) algorithms. Using a real-world dataset that consists of thousands of legal documents having complex language related to legal cases poses a challenging natural language understanding task by applying various textual features, deep features, and advanced sentence embeddings. The results reveal that the ensemble learning model of Extremely Randomized Trees shows better results with 89% accuracy, as it aggregates the results of multiple decorrelated decision trees to enhance predictive accuracy and control over-fitting. However, the best results of 96% are achieved with sentence embeddings. Sentence embeddings with Long Short-Term Memory (LSTM) networks are highly effective in Natural Language Processing (NLP) due to their ability to capture complex semantic and syntactic information within text.

## Introduction

Legal issues are core to the existence and efficiency of any civilization since they determine the parameters of operation for the members of that society. Laws are rules by which human interactions are bound [1], this channel ensuring justice and order by offering the way conduct is expected, or the process that is followed in the case of a dispute [2]. Any society is likely to face anarchy, people’s actions would be uncontrolled and random, and most issues would not have any legal preliminary of resolution. In addition, laws have the responsibility of safeguarding individuals’ rights and freedom, causing social reform and equality by implementing resulting in historical wrongs and social gaps [3]. For instance, laws play a central role in eliminating social evils such as human trafficking and racial discrimination, thereby paving the way for a justice system that genuinely serves society [4]. In the long run, a sound legal regime does more than protect liberties and ensures people have the capacity through agencies to uphold the law so that the role of law in a democratic society is upheld [5].

Further, the possibility of applying machine learning to legal work can also have a transformational effect on the industry of legal research. Conventional legal research entails physical scouring of case law, statutes, and regulations, and is therefore very cumbersome and can be associated with a lot of human interface errors [6]. NLP is an area of machine learning [7] that can help lawyers through large numbers of legal databases because the techniques can tell the difference between old cases, appeals, and legal principles [8]. This is not only beneficial for the legal professions themselves, but it also increases the effectiveness and accuracy of an argument, thus making research much more thorough and less likely to miss something [9]. ML models are used to enhance the method of searching for legal information and delivering the appropriate decision or suggesting an appropriate legal regulation [10]

Apart from enhancing the efficiency of the performance in the sphere of legal activity, the use of machine learning can produce results in the spread of legal materials. Historically, legal assistance has only been limited to a few, or those who have the knowledge or money for help. This gap is nonetheless filled by the ability of AI to pursue legal processes and make matters such as legal consultancy and analysis of documents more easily accessible to a broader public [11]. For instance, an AI legal-bot or document analysis programs help clients get preliminary legal advice or the assessment of their rights or completion of elementary legal paperwork, or interpretation of legal terms, without the necessity of gaining high-priced advice [12]. They all can increase access to justice and therefore bring down the price of legal aid and provide fit technological tools to empower users [13].

The goals of this study are outlined as follows: Firstly, to develop an accurate and fast ML model for the classification of legal texts, mainly colossal case documents. The second objective is to compare the application of the two types of methodologies in feature-based traditional ML and DL methodologies to determine the best approach to apply in the classification of legal texts.

With the exponential growth of legal case data, legal professionals often face challenges in efficiently retrieving, understanding, and analyzing relevant case information. It is time-consuming and error-prone to manually process large amounts of legal text, and in particular to keep track of previous legal cases and complex arguments in cases [14]. To address these challenges, NLP and deep learning methods have shown promise in automating legal text analysis. However, many existing models are either too computationally intensive or lack domain-specific adaptability. This study introduces a lightweight *Long Short-Term Memory* (LSTM)-based model enhanced with advanced sentence embeddings to automatically understand and predict legal case outcomes. The model not only assists in case classification but also enhances interpretability for legal practitioners by capturing contextual legal semantics. In doing so, the study addresses a crucial gap in scalable and efficient legal decision support systems, offering practical utility in jurisdictions handling high volumes of cases. This study has significant applications in the automation of legal document classification within judicial systems, law firms, and legal research institutions, enabling faster retrieval, sorting, and analysis of case documents. It can also be applied in regulatory compliance and e-governance platforms to streamline legal workflows and support decision-making through intelligent document handling.

For this concern, the paper relies on legal case records downloaded from the *Australian Legal Information Institute* (AustLII), a well-respected open-access provider of Australian law materials. The dataset is a collection of judicial judgments that originated from the Australian courts and were extracted for the period 2006–2009. Most of the cases are public law decisions, including administrative as well as constitutional decisions. This territorial focus provides for a coherent legal vocabulary and procedural setting, causing the deep learning model to be designed and tested to be fit for the task in the Australian legal environment.

In this paper, it has been presented how the application of machine learning techniques has greatly improved the recognition and categorization of legal document classification, thus improving the effectiveness of legal activities. Preliminary experiments have shown that in the classification of legal texts, well-developed approaches such as LSTM integrated with sentence embeddings achieve high accuracy without much pre-processing of documents compared to traditional machine learning methods. In this regard, the main contribution of the present study is the combination of fine-grained sentence embeddings with a deep learning-based LSTM model for legal document classification. Whereas previous studies have used more traditional feature representations (e.g., TF-IDF) or isolated deep neural networks, they are not always able to fully represent the complex semantic and contextual associations within legal texts. On the contrary, the framework proposed utilizes sentence-level semantic representations to encode contextual meaning and models them effectively with LSTM to learn sequential dependencies in legal documents. This hybrid model will allow a better interpretation of the structure of legal language, and much better classification results will be achieved. Moreover, the explainability methods (LIME and SHAP) introduce further interpretability, which increases the appropriateness of the model to the real-world legal decision-support systems. The contributions are as follows:

- Classifying legal documents to handle the large amount of case information by integrating fine-grained sentence embeddings based on deep learning models increases the effectiveness of managing text and optimizes entries into proper classes in the legal context.
- Comparing conventional classifiers used in ML algorithms and ensemble learning methods as baseline models ensures the identification of the most useful strategies in the classification of legal documents.
- Achieving the highest accuracy of 96% with the proposed model, LSTM, when coupled with advanced sentence embeddings.

The rest of the paper is organized as follows: The Related Work section shares the analysis of existing studies based on various ML and DL approaches. The Proposed Research Methodology section provides comprehensive details of the proposed methodology applied in this study. The Results Analysis section presents the discussion of results achieved based on the applied models. The Conclusion and Future Work section presents the conclusions and future directions.

## Related work

This section covers recent studies based on AI legal affairs and deep learning, exploring word embedding techniques.

### Use of AI in legal affairs

There are various NLP methods that have been utilized for most of the legal documents, such as contracts, case laws, statutes, etc. Legal personnel deal with huge amounts of texts that are legal documents, and therefore, AI becomes useful in improving the way legal tasks are handled [15]. Early research brought out the prospect of AI technology replacing basic legal operations like document review and clause identification [16]. From BERT, legal texts have been fine-tuned for legal text analyses that include named entity recognition, contract novation clause classification, and semantic similarity analysis [17]. Many of these improvements have cut the cycle time and effort needed to legally analyze documents while increasing the quality and standard of the work. Contract review and management has received a facelift from the advancement of AI with the automation of text mining for relevant clauses, commitments, and risks. Furthermore, Kira Systems and LawGeex can use machine learning to analyze critical clauses and check for anomalies and compliance with certain laws [18]. AI-assisted solutions were unveiled that can decrease the human factor influence and speed up contract analysis by 80% [19]. In addition to that, AI contracting platforms can track contract specifics, due dates, and renewals in real time, improving organizational productivity. AI-based applications such as ROSS Intelligence and CaseText operate by presenting lawyers with results that include case laws and legal analysis based on natural language requests [20]. Recent research has tried to apply graph-based artificial intelligence models to analyze citation graphs and assess the outcome of cases to support legal reasoning and judgment [21].

Decision support systems developed from past case data to try to make future predictions have also been used in the prediction of judicial behavior and evaluating associated risks of lawsuits [22]. For example, using the AI technique, it is possible to predict the outcome of the case in the European Court of Human Rights with better than 75% outcomes [23]. Such systems empower lawyers with information that helps improve their ability to reach better decisions and, therefore, come up with proper legal approaches to suit various cases. However, the integration of AI into legal documents has the following ethical and regulatory issues. The problems like responsibility for algorithm selection, data protection, and causality of AI’s decisions have become critical and topical [24]. Further, a problem arises about how AI makes decisions and the subsequent lack of accountability on the part of technology. There have been specific demands for ethical guidelines concerning the use of AI and the creation of a regulatory framework [2].

The study was composed of labeled U.S. case documents, and they established that CNNs, when applied, had improved outcomes of deep learning and high classification accuracy of more than 90%, opposed to conventional classification techniques. This, to some extent, illustrates the ability to deliver great potential for the deep learning ability in dealing with the complex syntax of legal language [25]. Another work was devoted to the text’s classification using CRFs and CNN in the legal sphere. The authors stated that, in general, there was 90% accuracy in CNNs, but there was 97% accuracy in the cases, as stated by the authors, which proved that extensive care should be taken to choose the relevant algorithms depending on the nature of the legal texts to be classified [26]. This result highlights the need for tailored approaches in using ML methods in the classification of legal documents. An investigation introduced an ensemble approach, which combines CNNs with the aim of exploiting the benefits of both. This work led to improved classification accuracies and sturdiness. This shows that it is possible that hybrid model methodologies yield increased performance compared to stand-alone methods [27].

Furthermore, an empirical study pointed out that variability in form and terminology of different legal documents posed a major problem in the classification of legal documents. The researchers presented a new model, which entails the use of domain knowledge to enhance the results of the ML models, thereby enhancing classification [28]. This approach, therefore, emphasizes the need to have a contextual approach when developing AIOps for integration with ML for legal solutions. There is also discussion about explainable AI (XAI) in the classification of legal documents. A paper presented techniques [29] for making the models used in the classification of legal texts more transparent. For practitioners, this will enable them to better understand the decisions of the ML model and gain trust in the intelligent systems. Moreover, new developments in NLP technology [30] have allowed for text analysis to progress to a point where semantic similarity within the text of a legal instrument can be understood and compared. There are other chiefly intricate texts, including legal documents, where state-of-the-art techniques like BERT have been used to classify the texts with considerable precision, revealing how NLP models can be used in a legal context [31].

### Deep learning and word embedding-based studies

The results of recent studies show that deep learning and advanced embeddings work well for sorting legal cases in many different areas. In the beginning, the most common practice was using only static word embeddings and classical neural networks. For this example, the predictions of criminal charges and fines using text CNN, RNN, and DenseNet models were applied to BDCI 2017 data. The researchers observed that a deep TextDenseNet network was more effective than RNN models, making it clear that feature hierarchies learned can be better than manually designed features based on static embeddings but are less accurate than the newly introduced fine-tuned contextual embeddings [29]. Furthermore, the CNN classification model already led to greater results on Indian Supreme Court judgments, and more improvements were made using ensemble methods. They considered a variety of embeddings, such as domain-specific ones (InLegalBERT, etc.) and generative transformers, for labeling their case documents and achieved very high accuracy by combining an encoder and the T5 model together with SMOTE and feature selection. This proves that static embeddings only capture some patterns, whereas contextualized representations (like BERT and T5) provide much better performance in this task for legal text classification [32].

Some researchers also bring in information about law and how documents are linked into neural models. Several approaches include structural or relational methods to better support the reasoning found in law for classification [33]. In this area, graph-based approaches receive attention. A framework with multiple modules finds the key legal information in the facts and hands that information over to a predictor model. The improved model outperformed other techniques on the main Chinese legal benchmark, proving that including legal features is beneficial. Some scientific investigators take advantage of unlabeled case materials for learning [34]. Furthermore, the PILOT approach starts by finding similar cases, uses them for prediction, and shows much better results than when previous information is disregarded. By using this strategy, common law’s unique issues are addressed (finding and making sense of previous rulings), unlike the earlier techniques made for civil law. Other approaches to classification arrange and organize parts of legal judgments. The authors apply a BiLSTM to analyze court sentences in child custody disputes, identifying sentences based on their role as either the request, decision, or legal reasoning. Their model would allow legal analysts to quickly find the crucial parts by spotting lengthy rulings with an F1 score over 0.90. It means deep models are not just forecasting court outcomes but can additionally spot legal issues and assign rhetorical roles in the judgments [35].

By comparison, using traditional embedding models such as Word2Vec or GloVe with simple classifiers performs much more comparatively low. An optimum RNN could not perform as well as transformers in making judgment predictions. Generally, the sector now mainly uses contextual embeddings, and today’s studies focus on tweaking or enhancing these for law-related details. Recent observations show that while small, fine-tuned models are good at solving clear tasks, they provide stronger, ready-to-use performance on complex classifications and need fewer large data sets [36]. In essence, recent research covers deep models for specific tasks as well as models that can be easily transferred across many tasks. The greatest success in case classification by law has been achieved by adapting modern NLP approaches to legal data, resulting in far better descriptions of different topics, outcomes, and other features in law [37].

Recent research on research paper classification shows the increase in the importance of metadata and semantic representations as the means of overcoming the drawbacks of traditional methods. In [38], the authors highlighted the limitations of single-label and multi-label classification techniques with the help of Word2Vec to obtain and use semantic and contextual information, which leads to better results on popular datasets without using manually determined thresholds. In a similar way, [39] focused on the importance of classification based on metadata and showed that using title and keywords together substantially improves performance in comparison to either feature, and that classical models like Random Forest, K-Nearest Neighbor, and Decision Tree provided good F-measure performance. In the same line of thinking, [40] took the next step by combining high-level semantic modeling with BERT to create high-dimensional contextual embeddings from metadata attributes, then optimizing them using genetic algorithms to minimize dimensionality and computational cost. The experiment also confirmed that the best metadata sets, especially title and keywords, are always better than the other sets of features. All these pieces illustrate the transition of the traditional statistical representations to semantic-rich embeddings and optimized use of metadata to enhance the performance of research document classification.

Despite the advantages of applying AI to examine legal cases, there is a need to acknowledge the limitations of using these methods. There are privacy risks related to sensitive legal information being processed, concerns about the reliability of model predictions in high-stakes legal settings, and the problem of AI hallucinations—where models may generate plausible but incorrect or unverifiable information. These challenges underline the necessity of an intelligent AI model that guarantees the confidentiality of the data, explainability, and accountability in making decisions, and validation ingredients for mitigating misinformation. Continuing work on using advanced approaches for further development and improvements in techniques for advanced NLP will probably lead to the legal domain, which will benefit the practice of legal research.

## Proposed research methodology

**Fig 1:**
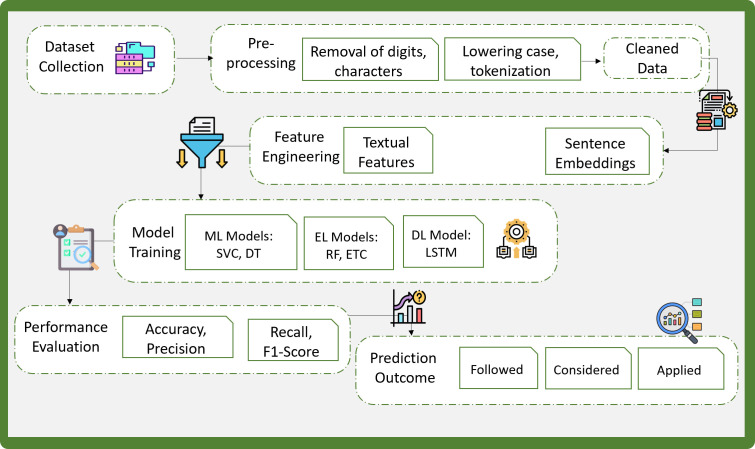
Steps of applied methodology.

A systematic approach based on the proposed methodology to classify legal documents is followed, as shown in Fig 1. The NLP pipeline is followed, considering well-established protocols, beginning with tokenization to preserve semantic boundaries before applying steps like punctuation and digit removal as per standard. This sequence ensures structural consistency and prevents distortion in sentence representation. A curated stop word list was used, initially based on the NLTK repository [41], imported globally online. This domain-aware approach to preprocessing enhances the contextual integrity of the data. Furthermore, the dataset comprises formal legal text excerpts, including court judgments and tribunal decisions, which have already been labeled into predefined categories.

### Data collection and preprocessing

The data set utilized in this study consists of 25,000 legal cases, with four primary columns: Case ID, Systemic Outcome, Case Title, and Case Description, freely available at an online repository [42]. To facilitate the classification of citations based on citation treatments and case outcomes, the dataset was constructed. A sample description of the corpus is presented in Table 1 to provide insight into the nature and linguistic patterns of the text used in model training.

**Table 1:** Sample sentences from the dataset showing case categories.

Case Category	Case Text
Followed	“The precedent set in Smith v. Jones was followed in determining the liability of the defendant.”
Followed	“In line with the ruling in Brown v. Board, the court followed the established legal principles.”
Applied	“The court applied the principles from Roe v. Wade to assess the plaintiff’s claim.”
Applied	“Applying the doctrine from Miranda v. Arizona, the defendant’s rights were evaluated.”
Considered	“While not binding, the court considered the findings from Johnson v. Texas in its deliberation.”
Considered	“The judgment took into account the considerations from Davis v. Monroe County Board of Education.”

**Fig 2:**
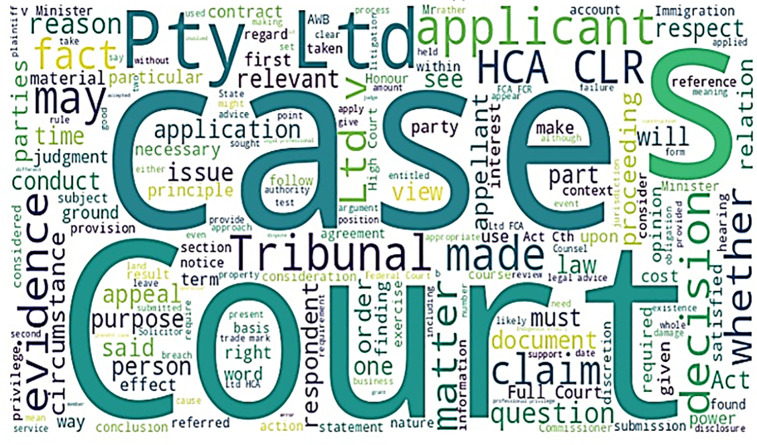
Most frequent words in dataset.

The most frequently used terms in the dataset appear as shown in Fig 2. A word cloud formed from major terms extracted from legal documents, which are prominently the key terms with more frequent appearance within court cases, judicial decisions, and legal proceedings. With highly characteristic words including ‘cases,’ ‘court,’ ‘tribunal,’ ‘decision,’ and ‘matter,’ it can be indicated that there is a very strong interest in legal adjudication, dispute resolution, and case law. Terms include ‘applicant’ (or ‘claimant’) as an official party in legal proceedings, ‘appeal’ or ‘respondent’ against whom the action is taken, ‘evidence’ as a legal document, ‘principal’ or ‘principle’ about which an issue is raised in a court ruling, and ‘provision’ or ‘law’ in the context of a legal ruling. This is an indication that the words HCA and CLR are abbreviations for certain legal reports or jurisdictions. In general, with the help of this word cloud, one can visualize well the critical terminology in legal texts for further analysis and legal document classification.

The text data in these columns was first reviewed for missing values and coherence. Due to the large amount of data, missing values were treated systematically and limited the entries with no textual information. In the textual data preprocessing process, some important steps were taken to eliminate noise and prepare the text for analysis. First, non-alphanumeric characters, such as special symbols, unnecessary punctuation, and digits, were eliminated using the following formal expression as in 1:


$$\begin{array}{c} T_{clean}=\left\{t\;\epsilon \;T\;\right|\;t\;\epsilon \;\left\{a,b,\ldots .z,\;A,B,\ldots ,Z,space\right\}\} \end{array}$$ (1)

where T is the original text, $T_{clean}$ is the cleaned text, t and represents each character in text.

In addition, raw words, such as those that are meaningless when the article is analyzed (e.g., ‘the,’ ‘and’ ‘is’), were excluded from the text [43]. A list of *stop words* was obtained from a standard repository and removed from each document using the following process, as in 2:


$$\begin{array}{c} T_{final}=remove\_stopwords(T_{clean}) \end{array}$$ (2)

Consequently, all the URLs and web addresses referenced in the texts of the cases under analysis were omitted because they are irrelevant to the legal context. This was done through regular expressions designed to match URL patterns, following in 3:


$$\begin{array}{c} T_{clean}=re.sub(r^{\prime }http[s]?://\${+}^{\prime },",\;T_{final}) \end{array}$$ (3)

### Embeddings and modeling

**Fig 3:**
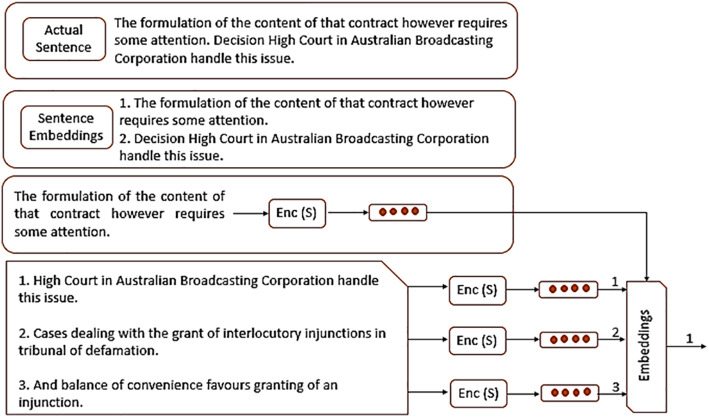
Working of sentence transformer.


In this paper, the application of expanding sentence embeddings with LSTMs in the classification of legal case text has been introduced. Each document of legal cases transforms into a vector into a dense real space with a fixed dimension, which is supposed to reflect meaning of sentences, $v_{s}\;\epsilon \;R^{d}$ where d is the dimensionality of the embedding space, working shown in Fig 3. This approach proves to be useful as the model is likely to capture on the context and semantics of the legal text needed to categorize the legal cases by their outcomes and citation treatments [44]. Further, used pre-trained *sentence embedding* models $S=\{w_{\text{1}},\;w_{\text{2}},\ldots .,w_{n}\}\;$ where to convert the text of each legal case into a relatively numerical format, formalized as in 4.

$$\begin{array}{c} v_{s}=f(S)=Embed(w_{\text{1}},\;w_{\text{2}},\ldots .,w_{n}) \end{array}$$ (4)

Where f(.) denotes the embedding function, and Embed is a transformation function that converts each word $w_{i}$ into its corresponding word embeddings $w_{i}\epsilon \;R^{d}$. The final sentence embeddings $v_{s}$ is obtained by combining the embeddings of individual words, often by averaging words, defined in 5.


$$\begin{array}{c} v_{s}=\;\frac{\text{1}}{n}\;\sum \overset{n}{\underset{i=\text{1}}{\mathrm{\backslash nolimits}}}w_{i} \end{array}$$ (5)

Recurrent Neural Network (RNN), i.e., LSTM, was then used on the embeddings of the input sentences. LSTMs are good for sequential data, for example, text, since they’re capable of tracking long-term relations and dependencies in a sentence [45]. This is particularly the case when the legal texts are being translated, and often, the meaning of a whole sentence greatly depends on previous sentences. This LSTM model had the ability to input the sentence embeddings from the text, acquire patterns and relational information from the text [46], and then classify each of the cases. At each time step t, forget gate $f_{t}$ controls the extent to which the previous cell state $C_{t-\text{1}}$ forgotten on the objective flow of bias term as, $f_{t}\;=\;\sigma \left(W_{f}\left[h_{t-\text{1}},\;v_{t}\right]+\;b_{f}\right)$. Then input gate $i_{t}\;$controls how much new information from the current input $v_{t}$ stored in the cell state as $i_{t}=\;\sigma \left(W_{i}\left[h_{t-\text{1}},\;v_{t}\right]+\;b_{i}\right)$.

**Fig 4:**
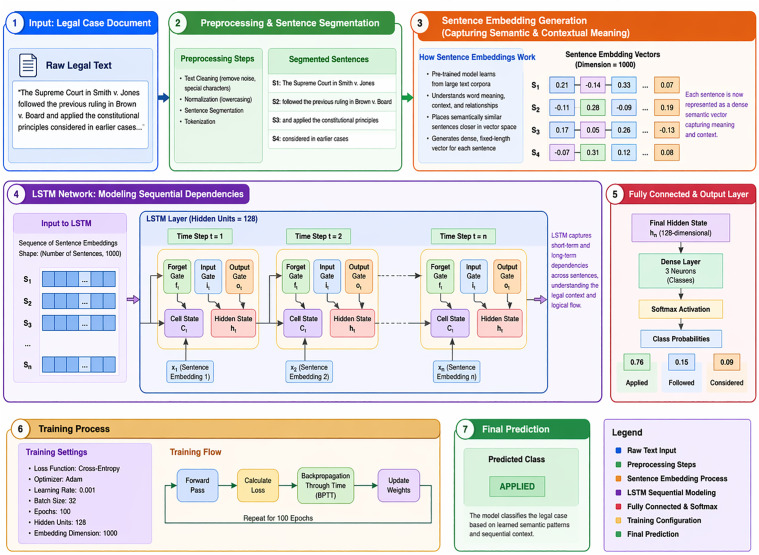
Architecture of Applied LSTM Model with Sentence Embeddings, showing the process of legal text into semantic vectors using embeddings, processing the sequence with LSTM to capture contextual dependencies, and using a fully connected layer to classify cases.

For new information, candidate cell $C_{t}$ potentially be added for the cell satte as $C_{t}=\text{tanh}(W_{c}\left[h_{t-\text{1}},\;v_{t}\right]+\;b_{c})$. This state further linked with the previous one to produce new cell candidate as $\begin{array}{c} C_{t}=\;f_{t}*\;C_{t-\text{1}}+\;i_{t}*\;C_{t} \end{array}$, to produce output as hidden state $o_{t}=\sigma \left(W_{o}\left[h_{t-\text{1}},\;v_{t}\right]+\;b_{o}\right),$ these states jointly using both semantic representation from the sentence embedding method and sequential dependencies from LSTM, as shown in Fig 4, the classification accuracy in legal case text would improve over simple sentence embeddings.

### Performance measures

To fully assess the performance of the proposed model, several evaluation measures were used, such as accuracy, precision, recall, and F1-score, as shown in Table 2. Although accuracy gives a general idea of the right predictions, it might not accurately represent the model in the multi-class classification tasks. Thus, precision and recall were adopted to measure the accuracy and fullness of predictions, respectively, and the F1-score is a balanced metric, as it is a combination of both precision and recall. These measures provide a more accurate and in-depth measure of classification performance, especially with complex textual legal data [47].

**Table 2:** Performance evaluation measures.

Metrics	Equation	Description
Accuracy	$\frac{TP+TN}{TF+FN+FP+TP}$	Evaluates the accuracy of the model as a whole by determining the ratio of accurately classified examples to all predictions.
Precision	$\frac{TP}{TP+FP}$	Represented as a fraction of the total instances that are predicted to be positive that are actually positive; predictive reliability.
Recall	$\frac{TP}{TP+FN}$	Quantifies how well the model is able to identify all the actual positive instances correctly; also referred to as sensitivity.
F1-score	$\frac{\text{2}(Precision*Recall)}{Precision+Recall}$	Offers an intermediate between accuracy and recall, particularly in cases where the classes are not equal.

### Model settings

To achieve reproducibility and transparency, Table 3 shows the overall experimental environment and the hyperparameter set employed in this study. The dataset contains 25,000 legal cases that are designed as a multi-classification problem with three classes: applied, followed, and considered. An eighty percent (80) to twenty percent (20) hold-out strategy was used to split the data into a training and test set to have a uniform evaluation protocol. To represent features, sentence embeddings were used to learn the semantic structure of legal text, with a 1000 input dimension. The suggested LSTM model will include a hidden layer of 128 units, and this layer will oversee learning sequential dependencies and contextual patterns in the embedded text sequences. The LSTM produces the hidden states, which are then fed to a fully connected (dense) layer, which projects the learned representations to the output space that represents the three legal classes. The output layer has a SoftMax activation to give class probabilities to be used in the final prediction. The model was trained at a batch size of 32 (100 epochs) using the Adam optimizer and a learning rate of 0.001. The cross-entropy loss function was used to guide the optimization process for multi-class classification. These hyperparameters were chosen to balance between the speed of convergence and generalization performance. Along with the proposed model, several baseline classifiers such as Support Vector Machine (SVM), Decision Tree (DT), Random Forest (RF), and Extra Trees Classifier (ETC)) were trained using standard hyperparameter settings in addition to the proposed model to allow a fair comparative analysis. The popular metrics were accuracy, precision, recall, and F1-score, which were used to measure model performance. It should be mentioned that the research is based on a supervised learning paradigm; thus, the parameters associated with active learning, including iterative labeling rounds, query strategy, and termination criteria, which are determined by annotation cycles, cannot be applied. The overall design provides a stable and strong experimental design to test the effectiveness of the proposed approach.

**Table 3:** Experimental setup and hyperparameter configuration.

Category	Parameter	Value
Dataset	Total Samples	25,000 legal documents
	Task Type	Multi-class classification
	Classes	Applied, Followed, Considered
	License	Apache 2.0
	Size	68.2MB
	Source	Kaggle
Data Split	Train–Test Split	80% Training – 20% Testing
	Sampling Strategy	Random
Input Representation	Embedding Type	Sentence Embeddings
	Input Dimension	1000
LSTM Model	Batch Size	32
	Hidden Dimension	128
	Learning Rate	0.001
	Epochs	100
	Optimizer	Adam
	Loss Function	Cross-Entropy
Baseline Models	SVM	Kernel = Linear, C = 1.0
	Decision Tree (DT)	Criterion = Gini, Max Depth = Default
	Random Forest (RF)	n_estimators = 100, Criterion = Gini
	Extra Trees (ETC)	n_estimators = 100, Criterion = Gini
Evaluation Metrics	Metrics Used	Accuracy, Precision, Recall, F1-score
Training Setup	Framework Type	Supervised Learning
	Active Learning Rounds	Not Applicable
	Initial Labeled Samples	Not Applicable
	Stopping Criteria	Fixed Epochs (100)

These parameters are defined to train the proposed model. The main aim is to develop an LSTM algorithm that effectively categorizes legal documents by leveraging sentence embeddings and the sequential characteristics of an LSTM network. This algorithm is intended for legal texts, which present highly context-dependent information, and encodes each sentence to a dense vector representation using the concept of sentence embeddings. These embeddings signify the meaning of the sentences and pass it to the LSTM network to understand the sequential nature between those two sentences. Finally, the last hidden state of the LSTM yields the document-level representation of the vocabulary that is then fed to a fully connected layer for classification of the input into some defined classes. The model, based on the algorithm, uses a cross-entropy loss function to optimize the prediction parameters via backpropagation. It is especially appropriate to use legal text analysis since such aggregation and context significance are vital in classification.

**Algorithm 1:** LSTM Model Training Process

1: **Input:** Legal document dataset with sentences represented as embeddings

2: **Output:** Predicted class labels for legal documents

3: **{*Sentence Encoding and Batch Preparation*}**

4: For each document in the dataset:

5:  Extract sentence embeddings $E\;\in \;R^{n*d}$

6:   Batch embeddings into B−sized groups for processing

7: ***{LSTM Forward Pass}***

8: For b=1 to |B|:

9:    For each sequence in batch b

10:     Initialize hidden state $h_{\text{0}}$ and cell state $c_{\text{0}}$

11:     For t=1 to n:

12:      $\;h_{t},\;c_{t}=LSTM(E_{t},\;h_{t-\text{1}},\;c_{t-\text{1}})$

13:     End For

14:     Extract the final hidden state $h_{n}$ for classification

15: End For

16: ***{Classification}***

17: For each document representation $h_{n}$:

18: Pass through the fully connected layer $y=softmax(Wh_{n}+b)$

19: End For

***20: {Loss Computation and Weight Update}***

21: Compute cross-entropy loss $L=\;-\frac{\text{1}}{\left|B\right|}\sum_{i=\text{1}}^{\left|B\right|}y_{i}\text{log}({\dot{y}}_{i})$

22: Back propagation gradients though model

23: Update weights $\theta \leftarrow \theta -\eta {△}_{\theta }L$.

24: End For

***25: {Prediction}***

26: For each test document:

27:    Repeat steps 5–19 for prediction

28: End For

## Results analysis

This section presents the results of the experimentation based on textual and deep features using various ML and DL approaches.

### Results with ML models

The performance results of the four models averaged in the following analysis clearly depict a significant difference among the models in terms of their ability to classify the legal cases. The SVC reveals very reasonable and balanced grades in all evaluated criteria, such as accuracy, precision, recall, and F1-score, which is 86%, reflecting its ability to make correct classification, as displayed in Table 4. Regarding the DT model, the accuracy, precision, recall, and F1 score were 81%, which, like SVC, is also relatively reliable with its predictions. In terms of the RF model, the accuracy is 84%, which is best at identifying positive cases. However, the ETC model stands out from all others with total accuracy and a throughput of 89% among all metrics, indicating the best balance and high accuracy in all criteria. The stable high accuracy in all reported criteria suggests that it is the best model for this data set.

**Table 4:** Machine learning results in percentage.

Model	Accuracy	Precision	Recall	F1-Score
SVC	86	86	86	86
DT	81	81	81	81
RF	84	88	84	83
ETC	89	89	89	89

**Fig 5:**
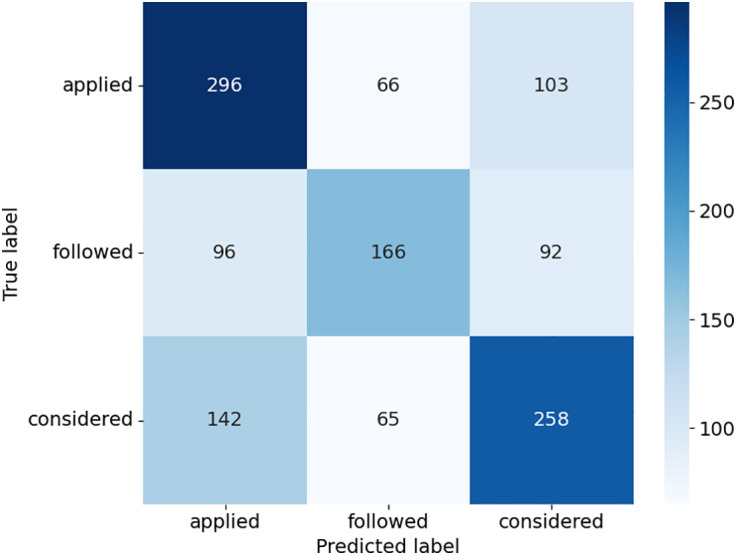
Confusion matrix of SVC model.

**Fig 6:**
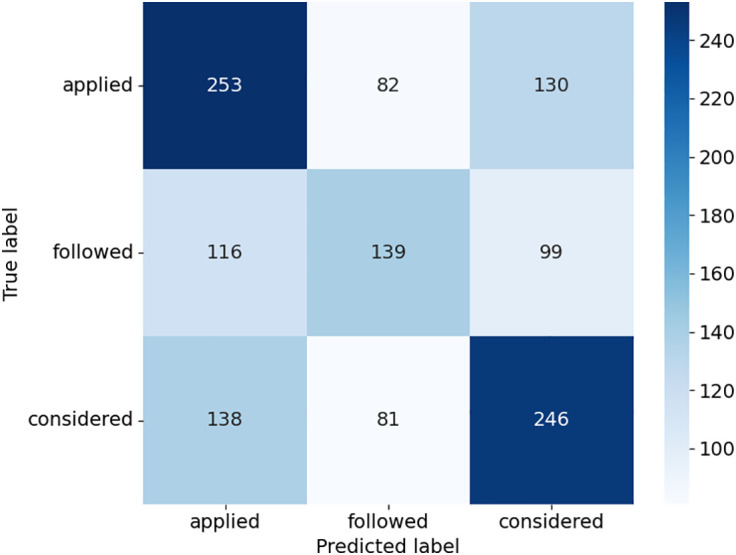
Confusion matrix of DT model.

**Fig 7:**
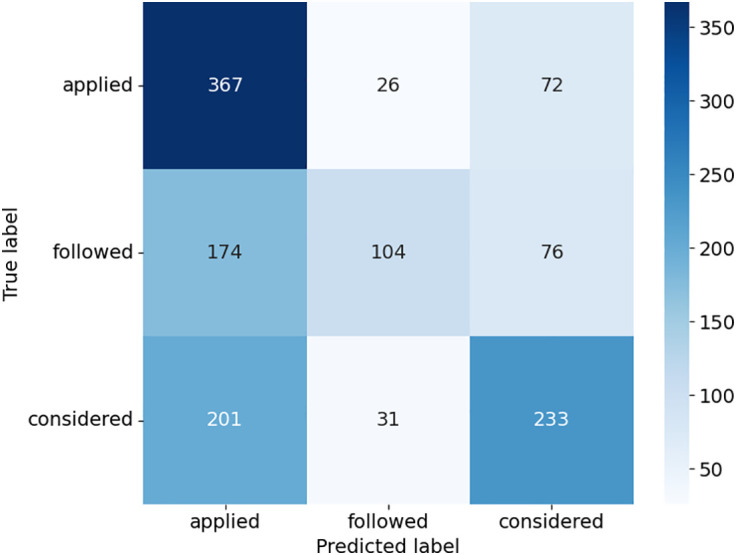
Confusion matrix of RF model.

**Fig 8:**
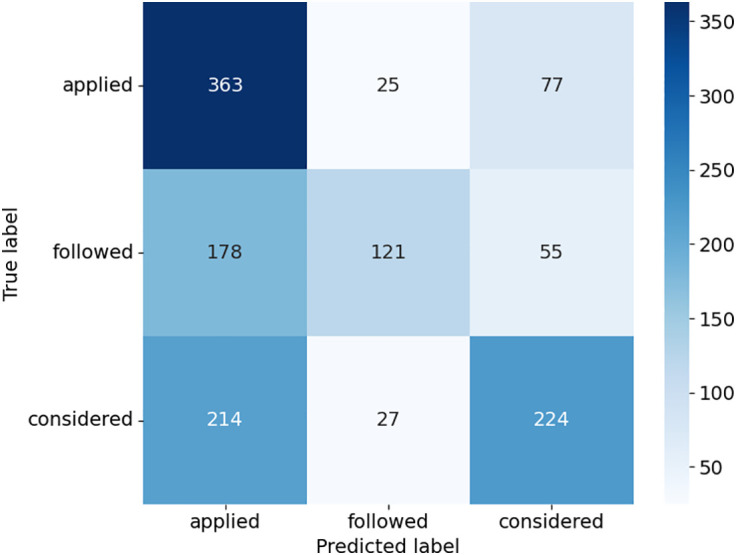
Confusion matrix of ETC model.

The confusion matrices provide detailed insights into how the models performed across the three case outcome classes in Figs 5–8. Consequently, this study uses the terms “applied,” “followed,” and “considered.” SVC in Fig 5, as presented with lower misclassifications among models, presented almost equally improved predictions. For example, there was a higher accuracy of the “considered” class with 258 true positives, but some confusion with the “applied” and “followed” classes. DT in Fig 6 had a relatively balanced distribution of the probability predictions; its misclassification rate is relatively higher in all classes, especially for the “followed” and “considered” ones, which suggests that it might be overfitting on some patterns. RF shown in Fig 7 was accurate in identifying “applied” (367 true positives), whereas there was a tendency to misclassify “followed” into other classes, hence the lower recall. ETC presented in Fig 8, though it uses the same structure as the RF, lost the capability to distinguish between classes, as can be seen from the confusion matrix, where Low, Medium, and High classes were interchanged more frequently. Overall, ensemble learning models (RF and ETC) are better than individual learning classifiers (SVC and DT), with ETC having the most accurate predictions overall, which demonstrates that ensemble learning is a stronger choice to learn with text in the legal field.

**Fig 9:**
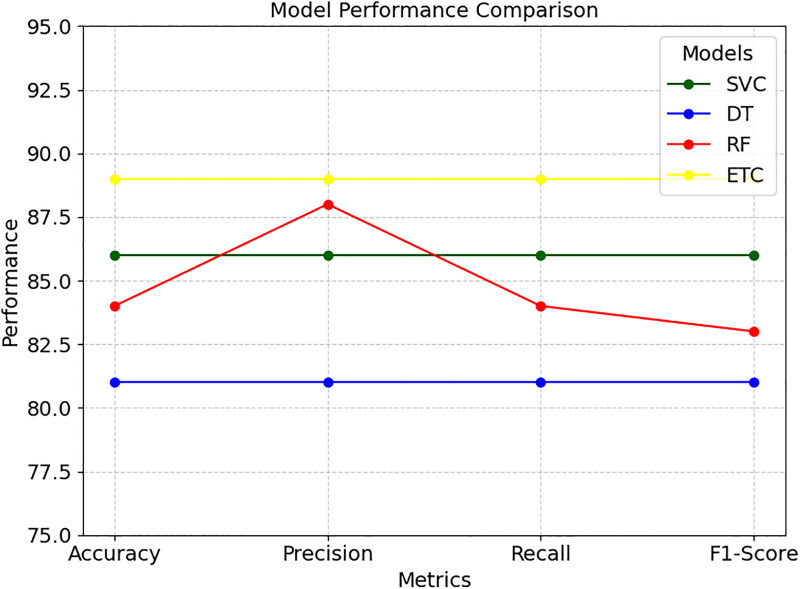
Comparison of all applied ML models.

In all terminology models, the ETC takes the superior performance among ML and Ensemble models in terms of accuracy, precision, recall, and F1-score, recorded at 89%, as shown in Fig 9, which is the highest among all the various models and thus qualifies it as the best model for this kind of classification.

### Results with DL models

**Fig 10:**
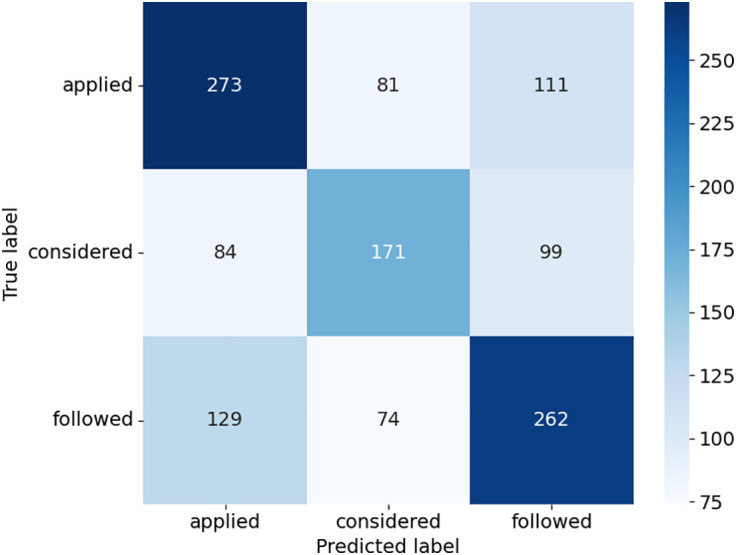
Confusion matrix of LSTM model.

The results of the LSTM model with Sentence Transformer highlight its strong ability to fit the training data but reveal significant challenges in generalizing to unseen cases, as displayed in Table 5. They also posed a very low loss equal to 0.13 with very high accuracy, precision, recall, and F1-score of 0.96, which demonstrated that the training model identified patterns in the training set effectively. On the other hand, the validation gave a loss of 1.79, and the performance metrics of accuracy were 0.54. The confusion matrix in Fig 10 further analyzes the performance of the model on each of the classes. For the “followed” class, the model was able to predict 262 of the correct classes that belonged to the “followed” and showed relatively poor performance while classifying the other classes.

**Table 5:** Results with deep model in percentage.

Metric	Train	Validate
Loss	13	17
Accuracy	96	54
Precision	96	54
Recall	96	54
F1-score	96	54

**Fig 11:**
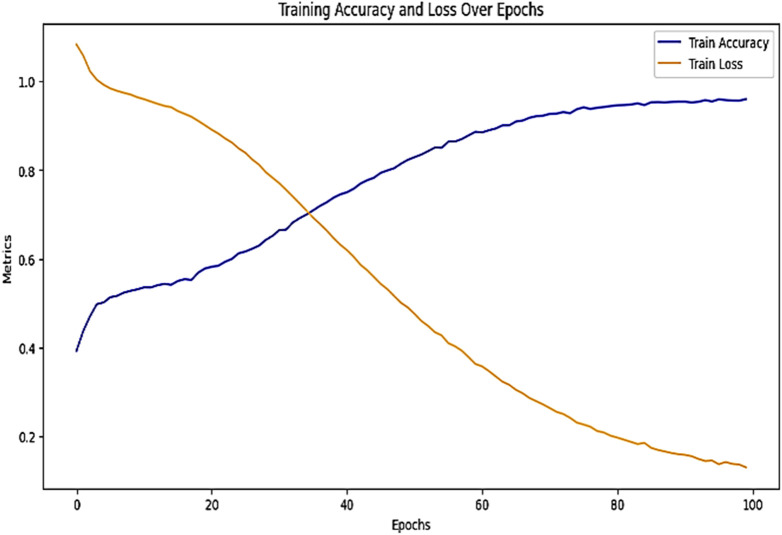
Training graph of LSTM model.

In the “applied” cases, 273 were correctly classified, although a good number of them were classified as “considered” or “followed”. Likewise, while for the “considered” class the model correctly classified 171 cases, as can be observed, the number of cases misclassified into the other two classes tells a different story. Such an aspect shows that although the model has the capability of making predictions, it does not differentiate between different classes, which creates confusion. The training graph in Fig 11 underpins these observations with increased training accuracy and the corresponding decreased training loss over epochs, indicating the good learning of the function on the training dataset. This difference in the training and validation accuracy means that a model has overfit, that is, it has built into its work only the training dataset, and the function does not generalize any information to other cases.

## Discussion

Over the past few years, the use of ML and DL used for legal document classification, due to the requirement to automate and speed up legal decisions. However, since statutes and precedent cases are typically long, dense and highly context dependent, the performance of classification-based models depend significantly on the quality of features and the degree of semantic information which they capture.

Through the comparative study of previous works, the significant difference of the performance of all previous works, mainly due to different model design and feature representation. For example, 91% and 92% accuracy was obtained with LSTMs and GloVe word embedding [44] and SVM classifiers with handcrafted textual features [45]. Although these methods successfully achieved good classification results, they used static word embeddings or hand-designed features (heuristic rules), which cannot capture deep contextual semantics in legal documents. The current paper also builds on this work, using sentence-level embeddings that capture wider semantic and syntactic relations to improve a model’s understanding of complex legal language.

Firstly, the models RF and Bi-LSTM mentioned in [6,46], and [47] were considered to have comparatively lower accuracy, amounting to 81% − 85, due to several reasons. Specifically, the RF model with the Bag-of-Words approach [6] was unable to maintain the contextual and sequential information retention, ultimately leading to fragmented feature representation. Moreover, the Bi-LSTM models from [46] and [47], even with their sequential design, adopted special embeddings obtained from Law2Vec, Word2Vec, and NER that might not generalize well across diverse legal document structures. In most cases, these features introduce a focus on domain-specific vocabulary without fully encoding the inter-sentence dependencies critical for understanding the legal rationale patterns. In contrast, the LSTM model from this paper achieved 96%, the best performance among those models, due to sentence embeddings integration that substantially enriched the semantic representation of the legal texts. Sentence embeddings do not operate on the simple word embeddings or BoW level of meaning but take meaning directly from the clauses and even documents, capturing various latent features and cross-sentence dependencies, which may prove critical for the legal-oriented classification tasks. Finally, the pre-processing pipeline in this paper includes domain-specific text normalization and cleaning, which became transformative in reducing noise and enhancing the input data quality that, in turn, resulted in the model’s performance increase. Secondly, due to the structured experimental set-up, this paper was able to afford a proper comparison between traditional ML, ensemble, and deep learning-based models. With the normalized evaluation and testing conditions, all discrepancies in performance are related to the model design and feature strategy rather than data set inconsistencies. Hence, the research results validate the importance of model-feature alignment in legal document classification and signify the outperformance of the LSTM-based model due to the richer understanding of the semantics and optimization of legal text processing.

**Fig 12:**
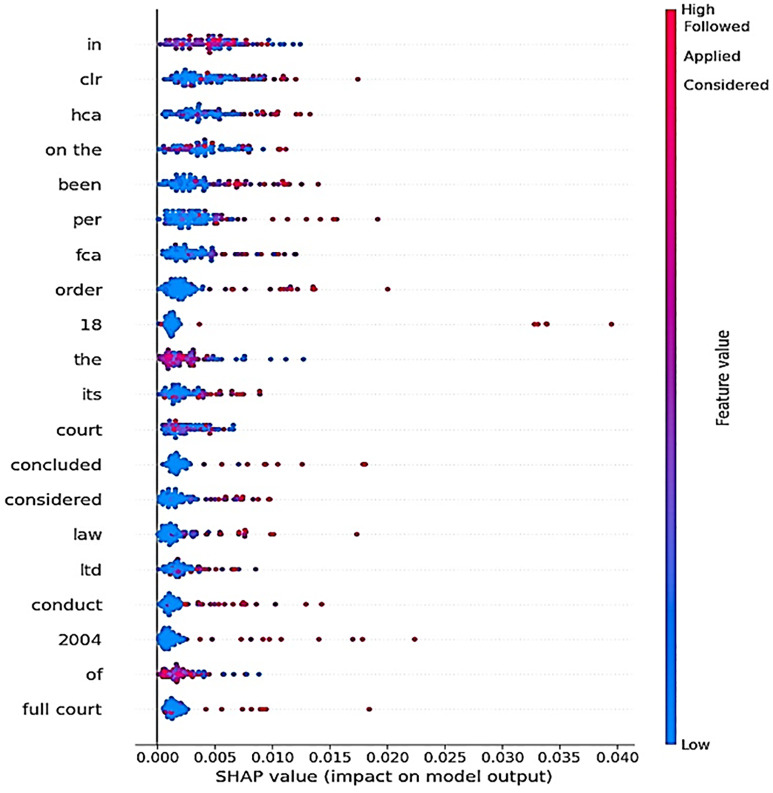
SHAP summary plot showing the impact of top textual features on the proposed model’s output.

This SHAP analysis visualization in Fig 12 shows how different word-based features contribute to the predictive strength of the model. The x-axis is the SHAP value, which is the feature’s contribution to the prediction, and the larger the number, the more the feature is pushing the prediction higher. The color scale changes linearly from blue to red, which corresponds to the characteristic value of the feature. Words such as “in,” “clr,” and “hca” exhibit a high holder of high SHAP values, which means a big positive impact of their presence on high values in the model’s decision-making. In contrast, terms including “court,” “concluded” and “law” prefer to have smaller SHAP values, suggesting a weaker or less consistent effect. In brief, this interpretation of the model indicates that it heavily depends on a set of legal terms and contextual tokens, especially, and both high-frequency terms play a relevant role in the separation of the classes. The attention network is capable of capturing subtle linguistic patterns that are important for legal case classification, as indicated by the different SHAP contributions across the important features.

**Fig 13:**
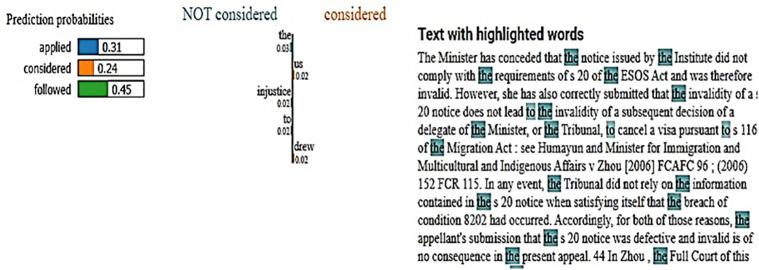
LIME explanation highlighting key words probabilities for classes.

**Fig 14:**
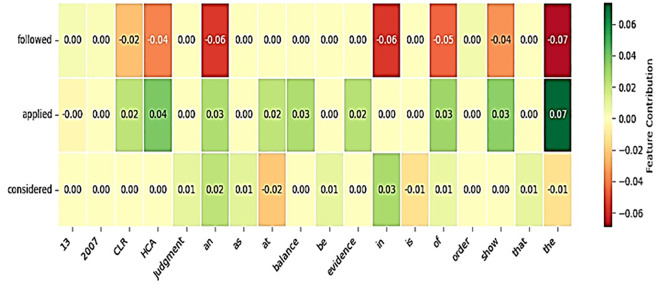
Combined LIME heatmap values illustrating feature contributions across all classes.

The LIME interpretation provides a detailed, localized explanation of the proposed model’s predictions for legal case classification. Fig 13 illustrates the prediction probabilities for the classes “applied,” “considered,” and “followed,” with the highest likelihood attributed to the “followed” class (0.45), followed by “applied” (0.31), and “considered” (0.24). The highlighted text in the legal document emphasizes key words such as “the,” “of,” and “to,” which LIME identifies as significant contributors influencing the model’s decision. Fig 14 complements this by showing a combined LIME heatmap for all classes, which visually encodes feature contributions where green indicates positive influence and red indicates negative influence on class predictions. Notably, words like “CLR,” “HCA,” “judgment,” and “order” show strong positive contributions towards the “applied” class, whereas common stop words like “the,” “that,” and “is” exhibit varied influence across classes, highlighting the subtle yet critical role of these tokens in the model’s classification process. Overall, LIME provides granular insight into how specific terms in legal texts drive classification, validating the model’s focus on legally significant vocabulary and contextual patterns within court case documents.

This paper is also novel because of a well-defined experimental setting, which allows fair comparison between the considered types of classifiers (traditional ML methods, ensemble classifiers, and deep learning models). Judging by consistent ranking and well-conditioned results, despite scores that vary more substantially than expected, differences in performance are mostly due to variance in model and feature strategies, rather than data discrepancies. Thus, the results strengthen the importance of model-feature alignment in legal document classification and demonstrate the ability of the proposed LSTM to improve state-of-the-art through enhanced semantic knowledge and tailored legal text handling. Implications. There are at least three significant implications of the findings in this study for the scientific and legal community. In terms of science, this work represents an additional step toward NLP and deep learning applications in the law domain, demonstrating the efficiency of sentence embeddings and LSTM networks for handling sophisticated and hard-to-discriminate legal documents with desired accuracy. Technically, the presented model offers legal experts a tool to automate the processes of document scrutiny and reviewing, to decrease the time and effort spent on document analysis, and to support them in making informed decisions. And the model’s effectiveness at reading legal texts can have a few positive implications for legal workflows, justice accessibility, and legal resource deployment.

There are several limitations to the present study, although the initial findings are promising. First, the performance of the model is very sensitive to the quality and the representativeness of the dataset for training. While the collection is tailored to cover a wide variety of different types of legal documents, it might be challenging to represent the great variety of language and style in the broader scope of law. Moreover, the feature space is text-biased, and it is interesting to see whether the performance of legal case classification can be further enhanced using multimodal data integration.

### Comparison of the proposed model with ML

Differences in effectiveness and response of the models are observed, as in Table 6, when comparing the ML models with the DL model. The specific analysis of the SVC model ranked well among the ML models. As for the LSTM model, it was able to train at a higher level of accuracy for the tuning data. This difference implies that the LSTM model exploits detailed features in the training dataset. In addition, the performance of the occurring ML models, primarily RF and SVC, was less effective for the overfitting session to both the training and validation datasets, as compared to the LSTM.

**Table 6:** Comparison of ML models with the proposed model (%).

Approach	Model	Accuracy	Precision	Recall	F1-Score
**Machine Learning**	SVC	86	86	86	86
	DT	81	81	81	81
**Ensemble Learning**	RF	84	88	84	83
	ETC	89	89	89	89
**Deep Learning**	**LSTM**	**96**	**96**	**96**	**96**

### Comparison with existing studies

The proposed LSTM model, combined with enhanced sentence embeddings, outperforms all the benchmark models in the classification of legal documents with an amazing Training accuracy of 96%. This result sets our proposed model as a promising improvement based on past techniques, as displayed in Table 7. Earlier works using conventional machine learning techniques, including RF and SVM, were indicated to predict specific contexts with accuracies of over 81% to 94%. However, these methods depend heavily on the handcrafted features and do not possess the ability to model the complex semantic structures, which are found in legal documents. While deep learning models such as BiLSTM have been considered in prior work, they have not yet been able to fully solve for the richness of legal document classification as demonstrated by our LSTM-based method. In addition, modern models, including BERT and T5, have promising performance across 79–91% accuracy.

**Table 7:** Comparison of proposed with existing studies’ results in %age accuracies.

Ref	Year	Model	Features	Results
[48]	2020	LSTM	GloVe	91
[49]	2021	SVM	Textual Features	92
[6]	2022	RF	BoW	84
[50]	2024	Bi-LSTM	Law2Vec	81
[17]	2024	LegalBERT	Contextual Embeddings	74
[51]	2025	Bi-LSTM	Word2Vec, NER	85
[31]	2025	BERT	Contextual Embeddings	89
[32]	2025	T5	Contextual Embeddings	93
**Proposed**		**LSTM**	**Sentence Embeddings**	**96**

Thus, the lightweight LSTM model focuses on the use of sentence embeddings, which enables a better understanding of the fine semantic features that the main aim is to capture to better comprehend the complex legal language and context. This capability not only outperforms traditional word embedding and LSTM-based work but also sets a new state of the art within the domain. This allows achieving a robust ability of mapping sequential dependencies and recognizing contextual meanings, which are especially important for the classification of legal documents – this work’s focus. Therefore, the proposed model makes it possible and shows the necessity to integrate the state-of-the-art procedures for further development of this research area.

## Conclusion and future work

The increasing complexity and volume of documents require enhanced approaches for classification and assessment, which greatly affect legal education and practice. Through this investigation, in this research, our findings related to the utilization of sentence embeddings and deep learning models to understand the possibility of automated classification of the texts of legal nature to improve the experience of legal research and learning. Study proves how these deep learning models, particularly LSTM with the sentence embeddings, can replicate training in data with a high training accuracy of 96%. On the other hand, there was better reliability and stability in model performance, especially in the ETC, with an accuracy of 89% in all four methods among ML models. This compares well with the effectiveness of ensemble-based approaches over deep learning algorithms in the current context. As shown by the results, complexity and generalization ability should be simultaneously considered, especially for legal document classification. Future directions should continue searching for ways to reduce overtraining in deep learning models, to use the combination of machine learning and deep learning techniques, or regularization and dropout techniques. Furthermore, incorporating methods such as citation analysis and using the best present-day embedding can also improve model performance to enable more effective assistants in law teaching and practice.
